# IP in Times of Climate Crisis – A Problem or a Solution?

**DOI:** 10.1007/s40319-022-01192-9

**Published:** 2022-05-02

**Authors:** Begoña Gonzalez Otero

**Affiliations:** grid.469479.1 0000 0001 1955 4116 Dr.; Senior Research Fellow, Max Planck Institute for Innovation and Competition, Munich, Germany

Addressing and working on the climate crisis is a vital issue for us all. Achieving climate neutrality as set by the Paris Agreement is the challenge of our times. The interdependence of the climate, ecosystems, biodiversity and human societies is more present than ever before. The effects of climate change and the urgency of the climate crisis are undeniable. Any increase in global warming will affect human health, with primarily negative consequences for food security, water supply and human security.

Debates abound about how climate change is a new issue or is enhanced by human activity, or is part of nature’s expanded cycle. In any case, this is not a new debate. As Klaus Bosselmann pointed out in his book *The Principle of Sustainability*, we can find reflections on sustainability in the context of forests and economic growth as far back as the Age of Enlightenment across Europe in the 17th to 19th centuries. The problem of our age is that we have waited until literally the very last minute.

The latest Intergovernmental Panel on Climate Change (IPCC) reports, presented at the end of February and early April 2022 and approved by all the United Nations Member States, are categorical: 30 months – that is the deadline for global greenhouse gas (GHG) emissions to fall drastically. Alternatively, humankind has three years left to peak GHG emissions. By 2025 a reversal will require immediate and deep cuts in emissions everywhere if we want to give the world a chance of limiting future heating to 1.5C above pre-industrial levels, so that the world could become “climate neutral” by 2050.

Despite the lack of a legal definition according to international law standards, becoming climate neutral is evident in practice. It means reducing GHG emissions to low levels and compensating for any remaining emissions. Compensation can occur through various offsetting measures, such as land reforestation. Likewise, a climate-neutral target is more ambitious than a carbon neutrality target. The latter targets only carbon dioxide, while the former includes methane, nitrous oxide and other gases. Thus, establishing “climate neutrality” as a climate policy goal worldwide, as the Paris Agreement did, entails a drastic change from previous international commitments. However, reaching climate neutrality is feasible but very hard. Limiting global warming will require significant shifts in the energy sector. The United Nations climate reports are very explicit about the need to rapidly phase out fossil fuel supply and demand: coal by 98%, oil by 60% and gas by 45% by 2050. This will involve a substantial reduction in fossil fuel use, widespread electrification, improved energy efficiency, and the use of alternative fuels. Such a reduction within this timeframe will require innovation at a scale and speed never before seen in the energy sector. On the bright side, we have witnessed massive innovation scales previously, such as the world wars, and in other sectors, such as the digital sector. What is undeniable is that we need a massive innovation effort in order to reach climate neutrality.

Therefore, the stated 30-month deadline comes with the need for a drastic change in innovation and economic growth models. Their enabling requires increasing adaptation and mitigation investments, policy instruments, the acceleration of technological innovation, and behavioural changes. Within this context, what role should intellectual property play? Intellectual property law (and competition law) are committed to providing investment incentives in research and development and to prohibit imitations without remuneration while retaining competitive pressure. The relationship between intellectual property and climate change is nothing but new. It has been the subject of critical discussions at the international level, and it has strong echoes of innovation. As Cicero said, “If you ignore what happened before you were born, you will always be a child”. Abbe E.L. Brown’s monograph from 2019, *Intellectual Property, Climate Change and Technology*, provides an excellent background on the international relationship between intellectual property and climate change.

If we go back to the 1992 Convention on Biological Diversity, Art. 16(2) and (3) stipulate that intellectual property access must be allowed, subject to conditions consistent with its adequate and effective protection. Furthermore, the Convention mandates that states cooperate in ensuring that intellectual property supports, and does not run counter to, the Convention’s objectives. And, in particular, that the implementation of the Agreement on Trade-Related Aspects of Intellectual Property Rights (TRIPS) does not result in intellectual property operating against the Convention (Art. 16(5)). This last complex relationship is also reflected in the 2010 “Nagoya Protocol on Access and Benefit Sharing”. As the Protocol states in its Annex “Monetary and Non-Monetary Benefits”, one way to reward those involved in providing and protecting raw resources is through intellectual property rights. Other methods include paying sample fees, researching funding, gaining access to datasets, and ensuring food and livelihood security.

It was not until 1997 that Art. 10 of the Kyoto Protocol recognized a possible conflict between private owners of intellectual property rights and state obligations under international agreements related to environmental protection and climate change. This provision requires states to create an environment that encourages the transfer of and access to environmentally sound technologies. For a decade, an extensive body of literature, including empirical studies and policy analyses, examined the extent to which that conflict was for real. As a result, states sought to create a post-Kyoto regime prior to the United Nations Framework Convention on Climate Change (UNFCCC) 2009 Copenhagen meeting. But that never happened. Such intervention seemed unjustified. Interference with the power of intellectual property rightsholders could be counterproductive by hindering the development of new technologies. Furthermore, since intellectual property could be merely a distraction, it was absent from the International Law Association’s 2014 “Declaration of Legal Principles Relating to Climate Change”, even if this declaration did not explicitly address technology.

Regardless, the debate surrounding the need for more significant intervention in the power of intellectual property with respect to climate change has continued. Some more recent examples include India’s ratification of the Paris Agreement in 2016, making clear the need for support from developed countries regarding access to technology. Similarly, in 2017, South Africa’s position on the implementation of the Paris Agreement stated “climate technologies need to flow, without hiding behind the issue of intellectual property rights”.

Interestingly, the United Nations report released in April 2022 includes, for the first time, a dedicated chapter on innovation, technology development and transfer (Chapter 16). It even contains a section on intellectual property rights and their impact on innovation (Chapter 16.4.6). Even if it mainly focuses on technology transfer (similar to the Copenhagen meeting, which led to the technology mechanism), it questions (again) whether patent systems promote innovation.

So, is intellectual property a problem or a solution? To answer this question, one must consider how innovation, social justice and the climate crisis connect to intellectual property systems. Intellectual property rights are increasingly being used for purposes beyond promoting innovation, e.g. as a commodity and object of investments, or merely strategic instruments. From a social justice perspective, innovations designed to reduce GHG emissions are still more expensive than those that pollute, because we do not factor the environmental-related externalities into the market price paid by consumers. As a result, cleaner innovation technologies suffer from “the green premium”, as coined by Bill Gates. He argues that policies should prioritize innovation in (energy) technologies with a low “green premium” and facilitate access, use and deployment as much as possible. However, as long as a ready market for energy-efficient technologies is not guaranteed, firms have few market-driven incentives to invest in R&D designed to reduce GHG emissions.

In this scenario, trade secrecy may become a talisman for preventing access to information rather than an incentive to explore new technologies further. Nonetheless, the intellectual property system offers tools and theories that could be helpful in providing access and use without having to turn our incentive systems upside down. Some examples are open-source access regimes, licensing pools, an articulated public domain, or the innovation commons described by Jason Potts, Dietmar Harhoff and others in *Social Welfare Gains from Innovation Commons: Theory, Evidence, and Policy Implications.* More drastic mechanisms, such as compulsory licensing or the drafting of new use exceptions, should be used with caution. The role of these tools may increase vis-à-vis traditional intellectual property rights in socializing innovation away from the pure (and legitimate) profit motive.

The main issue with clean energy innovation is not the intellectual property system but instead that it combines both a high technology risk and a high market risk. Thus, fostering this kind of innovation requires targeted public support different from that which we have seen in the pharmaceutical sector. It needs ambitious and flexible innovation-push regulations to create a more secure environment for clean energy innovation, as well as demand-pull policies to provide certainty regarding market opportunities for clean energy innovations by creating initial market niches. Furthermore, policymakers have a huge role to play in situations where network effects may impact the diffusion of innovation for cleaner energy technologies. Prior investments in polluting technologies and path dependency may challenge the transition to cleaner ones – especially in cases where the diffusion of cleaner technologies depends on an underlying infrastructure and competes with polluting technologies for which such infrastructure is already in place.

In line with the above, a recent study published in *Nature* reveals that governments spent at unprecedented levels trying to heave the world’s economy out of the recession caused by the Covid-19 pandemic. Yet, promises for investment in “build-back-better”-type measures that would counter climate change, such as public R&D spending in low-carbon energy technologies, or accelerating clean energy technology rollouts, have not been met. Furthermore, the paper finds that today’s state “green investments” are proportionately less than those that followed previous recessions. At the current pace, existing “green investment” rates will not suffice to reach climate neutrality globally by 2050.

In order for intellectual property to be a solution, governments worldwide must position their economies strategically to compete in a post-carbon world. This means public investment and fostering private ventures in low-carbon industries, building institutions to make economies more resilient to future shocks, and helping those who rely on fossil fuel-based industries to transition sustainably to new livelihoods. The building of a more innovative, sustainable and resilient economy requires its reorientation. The role of intellectual property as a problem or solution is very much up to us.

